# Cadaveric biomechanical assessment of different configurations for a novel pin and plate fixation method in distal humerus fractures

**DOI:** 10.1038/s41598-023-50976-7

**Published:** 2024-01-02

**Authors:** Kaveh Same, Alireza Hakiminejad, Amir Nourani, Mohammad Hossein Nabian, Mehdi Foruozesh, Reza Shahriar Kamrani

**Affiliations:** 1 Center for Orthopedic Trans-Disciplinary Applied Research, Tehran University of Medical Sciences, Shariati Hospital, Jalal Al Ahmad Highway., Tehran, Iran; 2 Department of Mechanical Engineering, Sharif University of Technology, Azadi St., Tehran, Iran; 3 Legal Medicine Research Center, Legal Medicine Organization, Tehran, Iran

**Keywords:** Fracture repair, Bone

## Abstract

Use of dual pre-contoured plates has been accepted as the treatment of choice in distal humerus fractures despite challenges especially in very distal or highly fragmented fractures. Aiming to improve results in such instances, our newly proposed method uses several K-wires fixated by a small reconstruction plate. Drawing on the results of previous finite element studies, the current study aims to compare the stiffness of three clinically common variations of this method using biomechanical testing in cadaveric humeri. 24 samples were divided into three groups and fractures were simulated. Groups I and II used 1.5 mm K-wires in differing configurations while 2 mm wires were used in group III. All samples underwent compression, anterior and posterior bending, and torsional testing as well as failure testing. Our results indicated that Group III had significantly higher stiffness in flexion, extension, and torsion (*p* < 0.05). In failure, group III had the highest mean stiffness in anterior bending and torsion (861.2 N, 30.9 Nm). Based on previous and current results, this new Persian fixation method, especially when implemented using 2 mm K-wires, shows promise in achieving suitable stability and may be useful as an alternative approach in complex distal humerus fractures.

## Introduction

Distal humerus fractures are among the frequent and challenging traumas in the upper extremity. Although mostly caused by high-energy injuries (e.g., falls, vehicle accidents and sports events), these fractures may occur in senior patients even by mild collisions and traumas^1–5^. Very distal fractures or those with high fragmentation are especially challenging to treat. Such fractures are particularly frequent in the elderly as lower bone mineral densities make them more susceptible to injuries with complex bone fragmentation^2,6,7^.

Several complications during treatment can cause discomfort and partial disability in the elbow joint^1–3,8^. Common complications are delayed union or non-union, acute neuropathy, joint stiffness, and a limited range of motion (ROM). Usually, treatment method is chosen based on the severity of the injury and the physician's experience and expertise, but it is known that non-operative methods (e.g., splinting and casting) are prone to a higher risk of complications^1,4,7^. So far, different techniques have been proposed for distal humeral fractures including open reduction and internal fixation (ORIF). Sufficient anatomical reduction and reconstruction of articular surface are prerequisites for proper treatment and adequate fracture healing.

From a biomechanical point of view, a proper fixation must provide a sufficiently rigid construct to prevent fragments from gross displacement and prepare suitable conditions for bone healing. Presently, anatomically pre-contoured plates are the most popular choice in the fractures of distal humerus and are usually implemented in bi-columnar configurations (i.e., parallel, and orthogonal). Different arrangements in this technic can provide suitable stability and while most biomechanical studies suggest using a parallel configuration because of a higher rigidity, others have reported identical outcomes between different configurations^2,4,7,9–12^.

According to notable clinical results, dual plating is considered to be the gold standard for many of the different distal humerus fracture patterns^12–14^. However, in case of comminuted low supracondylar fractures, the method is not utterly practical and there is a 35% risk for further post-operative complications such as loss of reduction, nonunion, and ulnar neuropathy^15–17^. Moreover, the plate contour does not always completely match the bone, especially on distal parts and, therefore, new, and novel surgical techniques should be considered for complex fractures.

In the early twentieth century, K-wires were the treatment of choice in many fractures, however, due to their low rigidity and high probabilities of non-union, they are no longer routinely used in adults and are mostly reserved for pediatric patients, where they have shown more acceptable results^1–4,8,12,18,19^. Still, their low cost, abundance and wide availability, and high potential for customizability turn them into an interesting choice as a permanent fixation structure should their weaknesses be overcome. The Persian fixation or pin and plate method was introduced by Kamrani et al.^20^ in 2011 as an innovative surgical approach for complex fractures specifically in the supracondylar region. The technique utilizes multiple K-wires with diameters of 1 to 2 mm inserted distally towards the lateral and medial epicondyles with the pin endings bent and secured with a reconstruction plate and screws on the posterior side of the diaphysis. This method was first performed on 19 patients with intra-articular fractures with an average age of 46. All cases were followed up for 12 months and the results showed satisfactory outcomes for both elbow and shoulder functions^20,21^. Also, two finite element (FE) studies have been carried out regarding the Persian fixation technique which investigated the biomechanical behavior of the Persian fixation technique under various loading conditions. Hakiminejad et al.^22^ modeled different configurations of K-wire number, diameter, and plate position to analyze their effects on fixation stiffness. Another FE study by Jitprapaikulsarn and others found their tested variation of the method to have overall comparable stability to the conventional methods except in internal rotation loading^23^. Both studies validated their models against experimental data and established criteria for judging fixation construct rigidity and reliability. Due to result of these studies and promising clinical outcomes, we assume that it is safe to conclude that the Persian fixation method has the potential to present a suitable stability for stabilizing distal humerus fractures.

Despite the efforts, in-vitro biomechanical assessment of Persian fixation method as well as choosing the most appropriate configuration remains to be investigated. The current study was designed to compare three of the more promising surgical configurations of Persian fixation (based on results from the FE studies) in various loading conditions. In the cadaveric biomechanical testing, stability of each arrangement is analyzed under axial, anterior bending, posterior bending, and torsion. Our objective is to find the most stable and robust arrangement. We hypothesize that although the rigidity of the pin and plate method is not necessarily higher than conventional methods, it is high enough to be considered especially for low supracondylar fractures provided that a suitable configuration is implemented by the surgeon.

## Materials and methods

### Specimens

A total of 24 fresh frozen cadaveric humeri were obtained from the forensics department for the study. Use of cadaveric specimens in this study was approved by Tehran University of Medical Sciences research ethics committee under the code IR.TUMS.MEDICINE.REC.1398.966. All necessary consents and permissions were also obtained through Tehran legal medicine organization for the sample procurement. Two of the specimens were female while the remainder were male. Age estimation was performed for each humerus using forensic landmarks. Average age for the specimens was 45.1 (standard deviation (SD) 9.1). All specimens underwent a CT scan (Siemens Somatom Emotion; Siemens, Germany) to check the bone for pathologies and for density estimation. The scans were made with a 2 mm slice thickness. Sample bone densities were obtained in reference to the Hounsfield unit values of a phantom via quantitative computed tomography technique (QCT). The average density in the samples was 1.124 (SD 0.093) g/cm^3^ and none of the bones had a density in the osteoporosis spectrum^24–26^.

**Figure 1:**
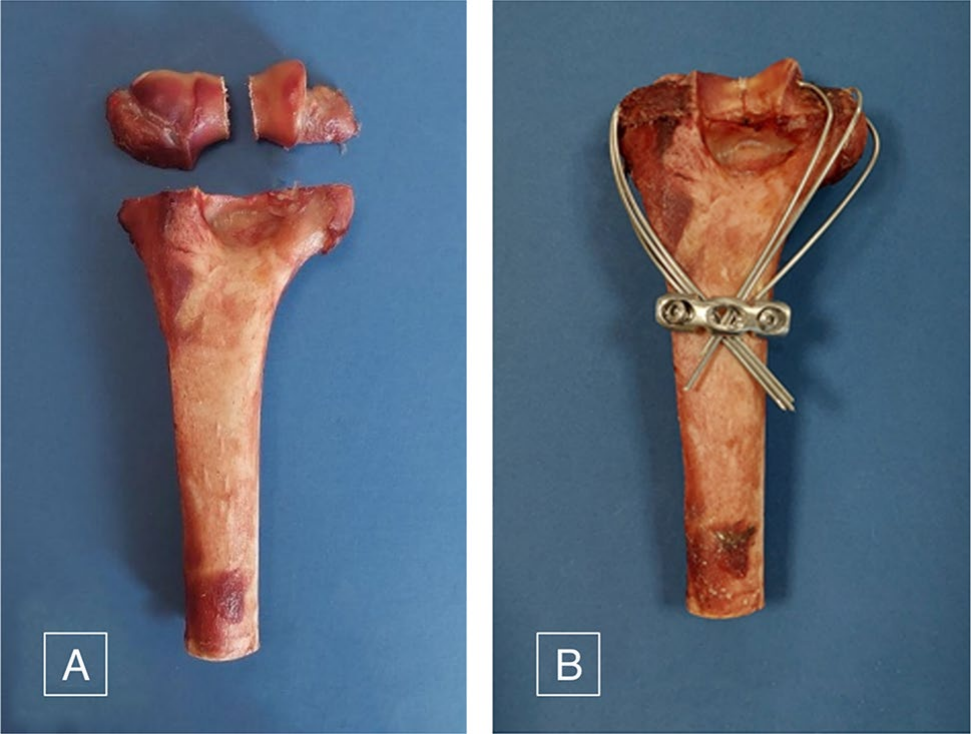
(**A**) The simulated fracture is depicted. The fracture type was chosen based on its high prevalence as the fracture 13C1.1 based on the AO classification. The horizontal cut is made parallel to the distal border of olecranon fossa. (**B**) Assembled fixation structure for a specimen in group I.

Afterwards, the samples were divided into three groups of eight. Groups were matched according to both age and density estimation. Furthermore, to assess the optimal number of specimens for each group, a series of pilot tests with identical implant configurations were performed and all of test results compared statistically. Average age and bone mineral densities of each group are listed in Table 1. The samples were stored in frozen conditions (i.e.,  − 20 °C), and the number of freeze-unfreeze cycles was tried to be kept minimum. Before further preparation, samples were thawed in room temperature for a sufficient time and were cut midshaft. The fracture type was chosen based on its high prevalence^27^ as fracture type 13C based on the AO fracture classification^30^. To simulate the fracture, a fretsaw was used to cut the bone parallel with the distal border of the olecranon fossa^28^. The distal fragment was further divided into two parts using the same saw^28^. No gap was created between the bone fragments to better simulate real-world circumstances (Fig. 1A).

**Table 1:** Properties of the three testing groups.

Group no	Age (year) mean ± SD	Bone mineral density (gr/cm^3^) mean ± SD	Diameter of K-wires (mm)
I	44 ± 8	1.13 ± 0.06	1.5
II	46 ± 6	1.11 ± 0.09	1.5
III	45 ± 4	1.14 ± 0.11	2

### Surgical method

**Figure 2:**
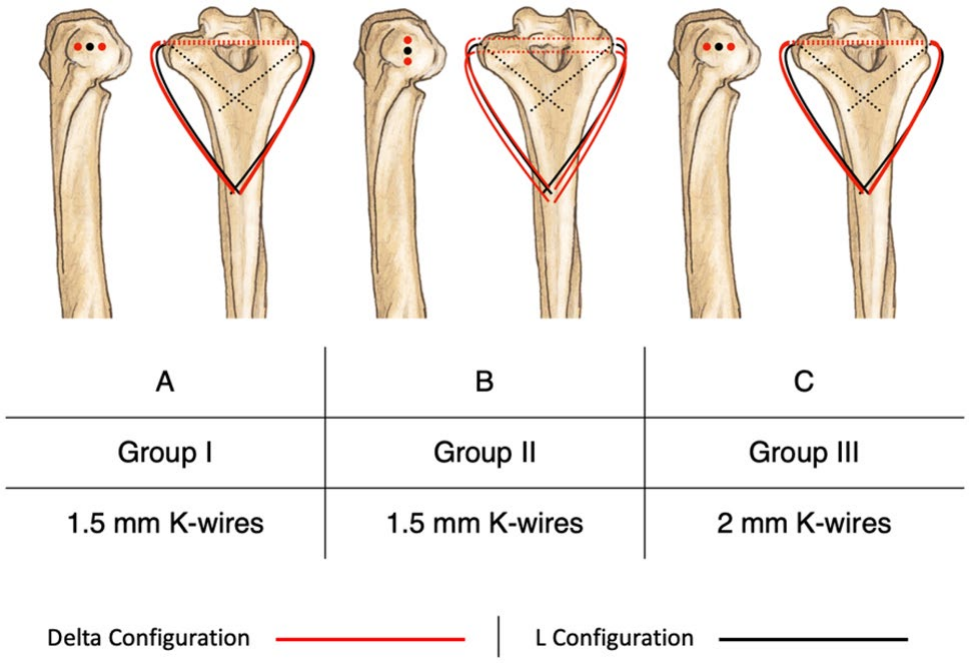
K-wire diameter and configuration in different groups. (**A**) Group I, four 1.5 mm pins were used to fixate the fracture fragments. The pins’ entry points into the bone were arranged in a horizontal line. (**B**) Group II, also using 1.5 mm pins but the entry points were arranged in a vertical line and (**C**) Group III, in this group, 2 mm pins were used in the same manner as the first group.

Three variations of the pin and plate fixation were designed. In each of the variants, four K-wires were implemented in the fixation construct so that two of the K-wires were arranged in L configuration and the other two in delta. In two of the variants (Groups I and II), pins with a diameter of 1.5 mm were used (Fig. 2A and B) while the third variant (Group III) utilized 2 mm K-wires (Fig. 2C). Groups I and II differed in the configuration of the pins in the fixation and their entry point into the bone while having the same pin diameters used. The third group had the same pin arrangement as group I but used 2 mm pins instead. The pin configurations for each group are depicted in Fig. 2.

In this study, all surgical procedures on cadaveric specimens were performed by the same surgeon and under direct supervision of an experienced orthopedic surgeon. This ensured that all procedures were performed accurately and safely, and that the results were reliable. Surgeries were performed in a random order, and all were conducted by the same surgeon using identical tools. First, the two distal fragments were fixated to the proximal bone using two pins (one per fragment). The K-wires were advanced until the pin tips protruded slightly from the bone cortex in the proximal. Next, two pins were inserted into the distal fragments in accordance with the experiment group’s configuration (see Fig. 2). Afterwards, all the pins were bent towards the posterior surface of the bone shaft and any extra pin length was cut. The pins were fixed in place via a curved three-holed 3.5 mm plate secured to the bone with two double-cortex, self-tapping, 3.5 mm screws. Plate curvature was adjusted for each sample based on the needed shape. Proper care was taken for the pins to protrude from under the plate a sufficient length to minimize the chance of pins dislodging from under the plate during testing. Screw lengths were chosen based on the need and according to bone shaft diameter. All surgical implants and tools were purchased from Atlas Bone Teb Co., Tabriz, Iran, and were licensed for use in human patients. Fixation structure for a specimen in group I is depicted in Fig. 1B.

### Sample preparation

**Figure 3:**
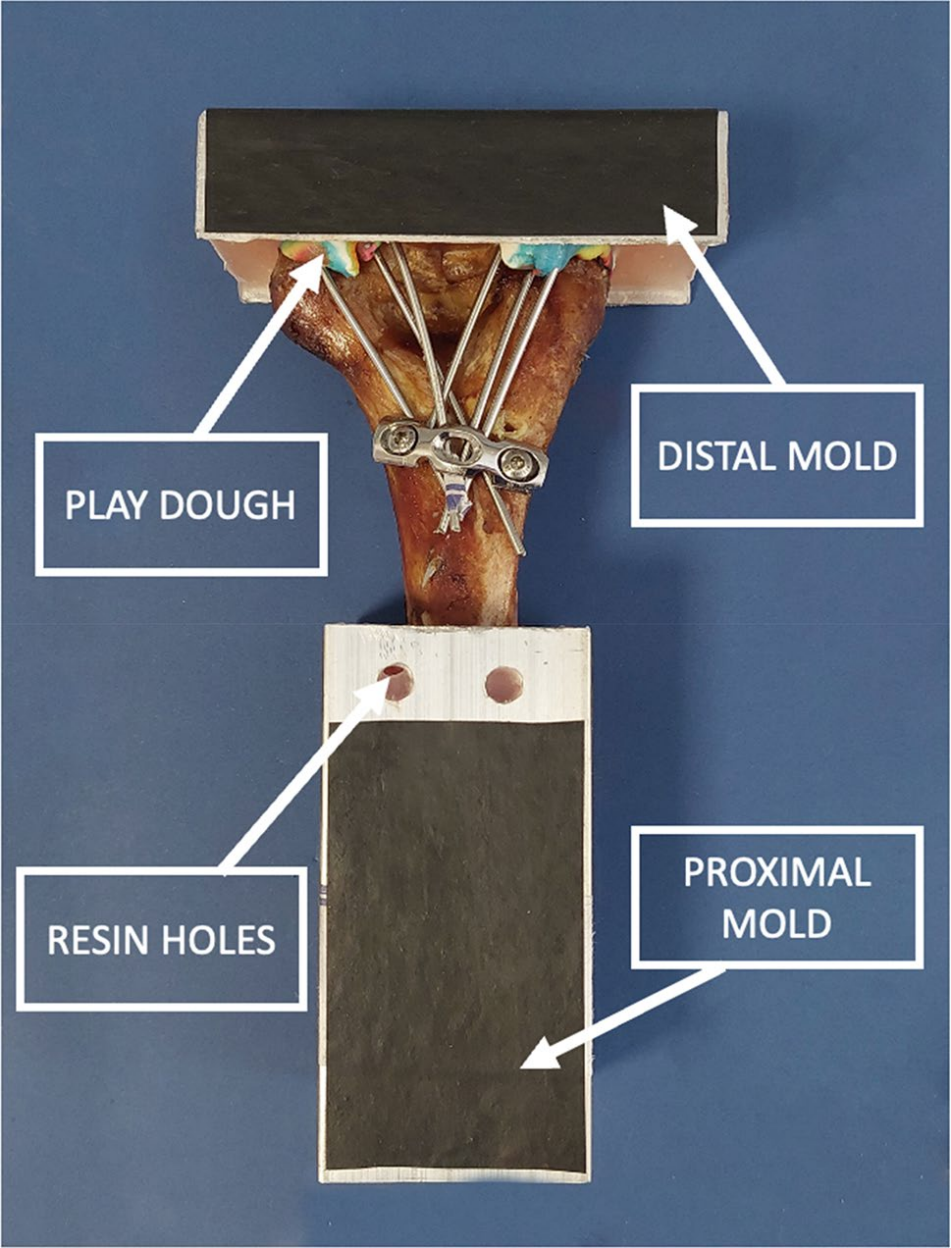
Potted sample in the proximal and distal aluminum molds. Note the play dough covering pins in the distal mold to prevent the implants from adhering to the resin. Also visible are the resin holes in the proximal mold implemented to ensure secure attachment of the resin to the mold.

After the surgery, all samples were potted in proximal (Fig. 3) and distal molds made of aluminum to prepare them for biomechanical testing. The samples were fixed in the mold with the use of a methyl methacrylate resin and cold-cure acrylic (Acropars Co., Tehran, Iran). In the distal mold, the exit points of the pins were covered with a non-drying play dough before the application of the resin to ensure non-contact between poly methyl methacrylate (PMMA) and k-wires and, thus, allow for possible pin movement or loosening during testing. Care was also taken for the resin not to cover any part of the horizontal fracture line. To ensure a secure fit between the resin and aluminum mold, holes were created in the mold to be filled with resin to eliminate any movements between dried PMMA and aluminum mold (Fig. 3).

### Biomechanical testing

**Figure 4:**
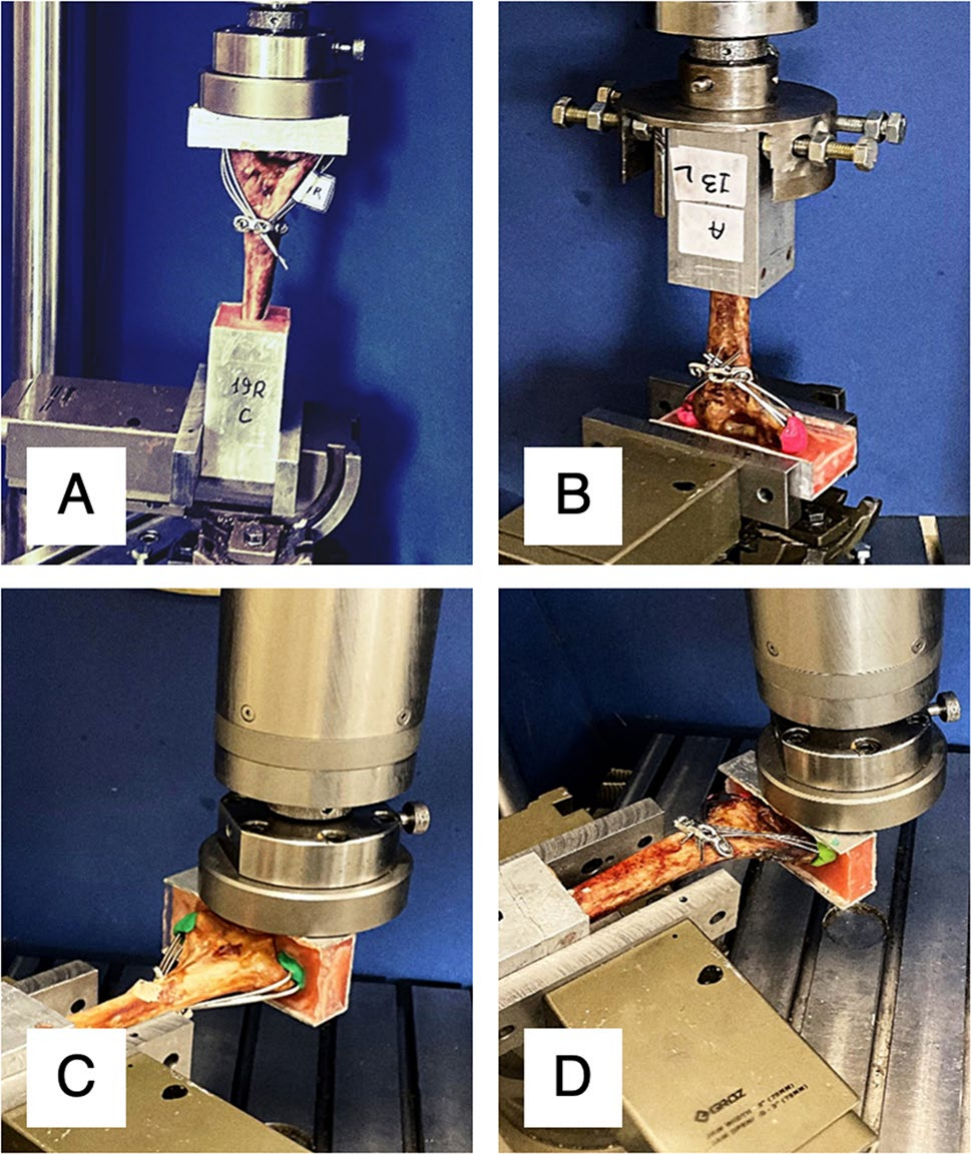
Testing setup for (**A**) Axial loading (**B**) Torsion (**C**) Posterior bending (**D**) Anterior bending.

**Figure 5:**
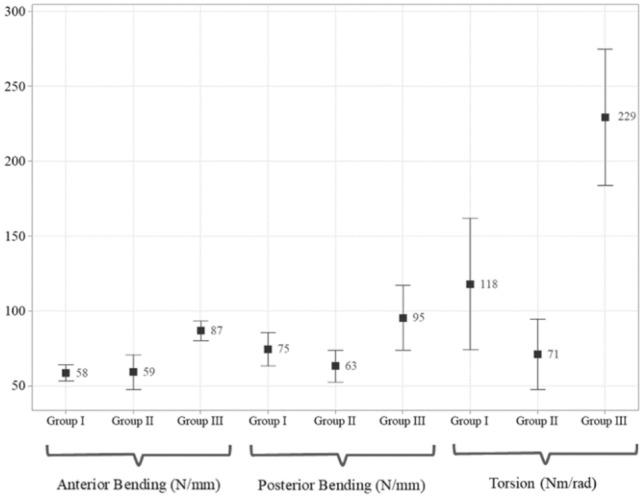
Stiffness values under cyclic anterior/posterior bending and torsion. The graph demonstrates the mean and standard deviation of measured values of stiffnesses in each group under different loading conditions. In anterior and posterior, group III had a modest dominance over other groups, while in torsion, group III provided significant additional stability.

First, each specimen underwent the same order of non-destructive tests, namely, compression, anterior bending (flexion), posterior bending (extension), and torsion, using a servo-hydraulic testing machine (Amsler HCT 25-400; Zwick/Roell AG, Germany). Testing setup for different loading conditions is demonstrated in Fig. 5. Loading ranges for axial loading, anterior/posterior bending and torsion were 0–100 N, 0–30 N and ± 2 Nm respectively^2,3,5–8^. Each of the mentioned loading conditions were conducted in a cyclic manner for 20 cycles at 0.05 Hz^5–8,13,14,18,19,29^. All tests were performed well within the elastic region of the samples based on the forces obtained from the literature as well as pilot tests performed prior to the study (Fig. 4).

Samples were fitted into the testing machine with the use of a fixture capable of adjustments in all three planes and therefore each sample’s loading surface was confirmed to be leveled to ensure a uniform force application on the samples during all bending and compression tests. A test-to-failure was performed on each sample after the completion of non-destructive cyclic tests; the force was monotonically increased until a construction failure occurred. Half of the samples in each group underwent a test-to-failure in torsion, while the other half were tested in anterior bending. These two modes were chosen based on their significance in the daily forces encountered by the elbow joint. During these tests, a breakage of implants or bone structure was considered as a sign of failure.

Data for fixation stiffness (N/mm or Nm/rad) was obtained over the linear portion of each curve (0–100 N for compression, 0–30 N for flexion/extension and  ± 2 Nm for torsion). Stiffness and rigidity for each test were evaluated using a one-way ANOVA between all groups. A value of *p* < 0.05 was considered statistically significant. All the tests, as well as treatment and handling of human remains were carried out in accordance with ethical principles and relevant guidelines and regulations.

## Results

### Stiffness in cyclic testing

Figure 5 shows interval plots based on mean and standard variations for each group under different loading conditions. The highest stiffness values of specimens in axial loading were obtained for Group I (1013 N/mm), but the difference with other groups was not statically significant (*p* = 0.201). For groups II and III measured axial stiffness were 672 and 542 N/mm, respectively. The highest stiffness for both anterior and posterior bending loads were observed for Group III. In anterior bending, Group III showed a nearly 50% larger stiffness with respect to both groups I and II (*p* = 0.001), which themselves had an almost same rigidity (*p* = 0.917). In torsional loading, also, Group III tests yielded a higher stiffness than that of Groups I and II by 94% (*p* = 0.001) and 222% (*p* = 0.000), respectively.

### Strength and failure testing

**Figure 6:**
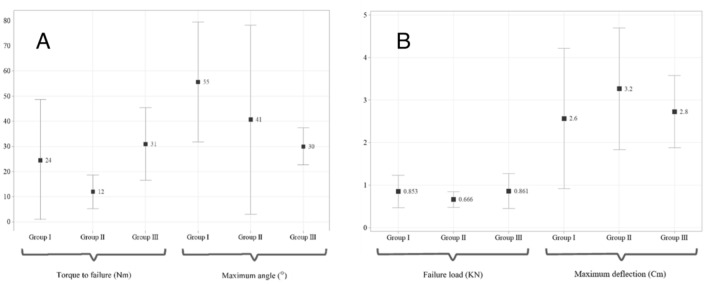
(**A**) Maximum torque and torsional angle until failure under monotonic torsional loading after the cyclic stage. There were no significant differences between the obtained result for each group. (**B**) Failure load and maximum deflection under monotonic anterior bending after the cyclic stage. Group II had a moderately lower failure load than other groups, but variations of the maximum deflection between different configurations were relatively minor.

After the cyclic phase, specimens in each group were divided into two groups to measure load and deflection upon fixation structure failure. Figure 6A shows the results obtained for monotonic torsion tests for different groups. In torsion, Group III showed a greater strength than the other groups, but the differences in maximum loading values were not significant (*p* = 0.074). Amount of maximum torsional angle was highest in group I and minimum torsional deflection occurred in group III. In bending, differences between strengths in different groups were statistically insignificant (*p* = 0.07). Moreover, alterations in maximum displacement as a result of bending force remained almost unchanged for all groups (*p* = 0.14). Detailed results for anterior bending failure can be seen in Figure 6B.

**Figure 7:**
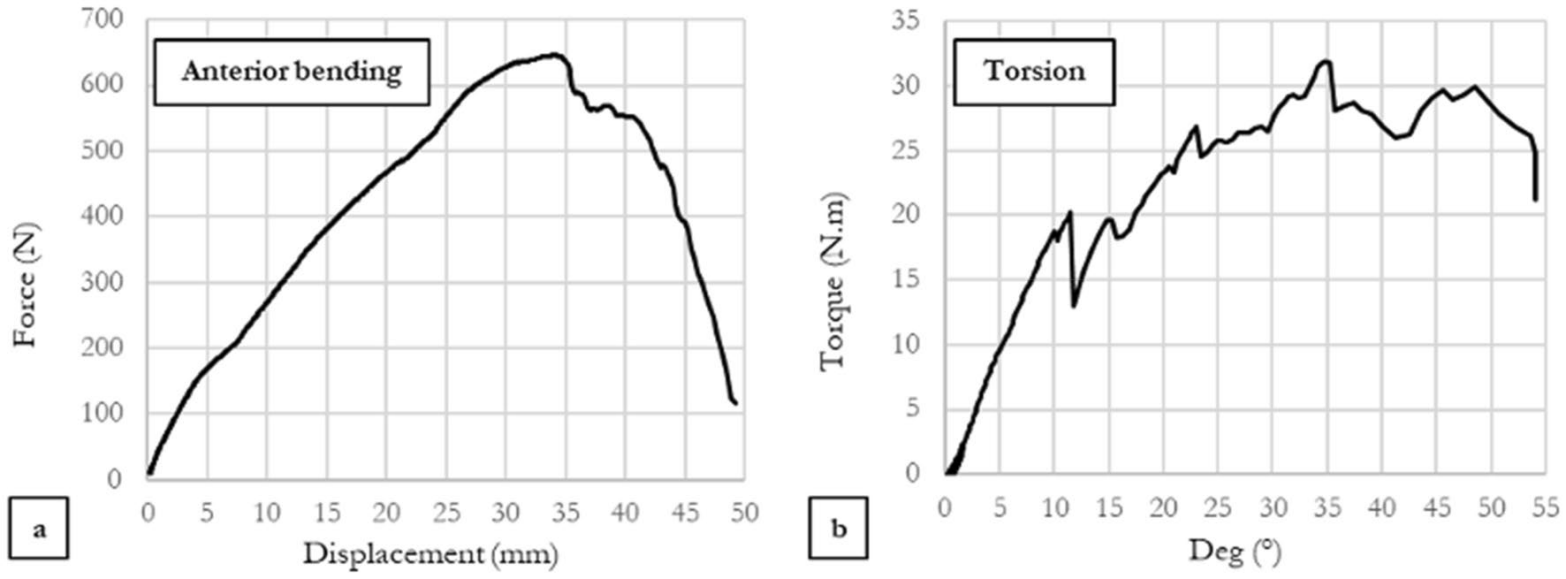
Failure graphs of two specimens under (**a**) Anterior bending, (**b**) Torsion.

Failure graphs for two specimens under anterior bending and torsional loading are illustrated in Figure 7a and b. It should be mentioned that most of the deflection in all specimens was observed in the non-linear (plastic) region of the force-displacement chart.

## Discussion

Persian fixation utilizes a set of K-wires to stabilize distal humerus fractures^20^. This technic has shown promising clinical results and stability especially in the fixation of very distal humerus fractures^20^. Furthermore, previous FE studies have found different variations of the method to have comparable stability and stiffness to those of the conventional methods of fixation^23^. Being a newly proposed method on one hand and considering the observed clinical results on the other, it is necessary to identify key biomechanical aspects of the method to better interpret and ascertain the observed outcomes and their relation to the exact arrangement of the fixation elements. Therefore, the present study, aimed to analyze biomechanical features of Persian fixation using cadaveric specimens.

In the present study, out of the three investigated pin configurations, groups I and III were included with the aim to ascertain the results of our previous FE study regarding the effect of K-wire diameter in the overall fixation structure stiffness^22^. Group II was added to explore a new factor (i.e., K-wire arrangement) and whether this factor can affect the structure stiffness. The specific K-wire arrangements were chosen via expert opinion. Our results indicated that stiffness of the fixation construct did not significantly differ between the three groups under axial loading, while in bending and torsion, fixations with 2 mm K-wires (Group III) had significantly higher stiffness than others. This is in agreement with our previous results. Patterns of failure in both torsional loading and anterior bending were similar for most samples in which distal fragments were sheared by the L-configuration K-wires. Such a finding emphasizes the importance of K-wires with L-configuration in the rigidity of the entire structure.

It is of note that in the fracture simulation in the present study, no gap was introduced between the two distal fragments and the proximal bone. This is in contradiction to some of the biomechanical studies assessing distal humerus fractures in which a gap was created between the distal and proximal fragments^2,4,6–8,10,11^ with the stated benefit of removing the effect of bone to bone contact from the measured stiffness. Still, other studies have been performed in the distal humerus without such a gap^30–32^, pointing towards differing opinions on the matter. Regardless of the pros and cons of such a gap between fragments, it is of note that a lack of space between bone fragments is the prerequisite for performing the Persian fixation method; i.e., if there is a gap between the fragments, this method will be impractical due to the significant elasticity of the fixation structure.

Also, according to our observed results, the pin and plate construct can resist loads far out of physiological range. This may be due to the K-wires ability to plastically deform much more than conventional implants^2,4,15^ (see Fig. 7). This in turn can lower the risk of implant breakage under a wider range of loading conditions. Furthermore, considering the much higher pliability of the K-wires compared to anatomical plates used in the conventional methods of fixation, the Persian method facilitates a much higher degree of customization in the fixation structure which allows the surgeon to tailor the construct based on the requirements of each fracture.

In addition, involvement of simpler tools and implants decreases the costs of operation as the devices used in the implementation of Persian fixation cost a fraction of the average cost of anatomical plates and screws used in the conventional method, resulting in a cost reduction of 90%^33^. Such an advantage may be of considerable importance where limited financial resources may be available. Lastly, use of simpler tools may also provide the surgeon with a more lenient learning curve when compared to that of the conventional method.

Overall, although the acquired results suggest that our proposed method is easily capable of withstanding daily forces, more studies, both clinical and biomechanical, such as a direct comparison between the biomechanical characteristics of the Persian and conventional methods are needed to further investigate this newly proposed method.

Finally, there are several limitations to our study. Due to the normal BMD of all cadaveric specimens, we were unable to analyze the effect of osteoporosis on the construct’s stiffness. Also, there was no control group of similar fractures fixed with dual-plating techniques for direct comparison of outcomes. Moreover, all cyclic testing was conducted within the elastic limits of the bone and implants and failure behavior in plastic region was only assessed post-cyclic testing. While our results may be reliable for this particular type of intra-articular fracture, further studies are needed to make a conclusive and general conclusion about the outcomes of our newly proposed method.

## Conclusions

The current biomechanical study investigated the biomechanical properties of three variations of a newly proposed method of fixation using K-wires and plate (i.e., the Persian fixation method) for the fixation of distal humerus fractures (fracture type 13C1.1 based on AO classification) in cadaveric humeri samples. Based on the results from previous FE studies and expert opinion, three variants of the method were chosen to assess the effects of K-wire diameter as well as their arrangement on the stiffness and failure threshold of the fixation construct. Our results indicated that the group using the thicker 2 mm wires showed significantly higher stiffness in anterior, posterior, and torsional loading which confirms previous simulation results. As for the arrangement of the K-wires, no significant relation was found between K-wire arrangement and the fixation structure stiffness. In failure, all groups had similar results and no significant differences were observed between them. The distinctive feature of this method was deforming in the non-elastic region which lowers the risk of implant failure in the postoperative period. Further research is warranted to assess the method characteristics compared to conventional methods of fixation for distal humerus fractures.

## Data Availability

The datasets generated during and/or analyzed during the current study are available from the corresponding author on reasonable request.
